# Determination of Offset Values in Binary Regression Models to Adjust for Misclassification Errors

**DOI:** 10.3390/ijerph23020220

**Published:** 2026-02-10

**Authors:** Moonseong Heo

**Affiliations:** Department of Public Health Sciences, 532 Edwards Hall, Clemson University, Clemson, SC 29634, USA; mheo@clemson.edu; Tel.: +1-(864)-455-1132

**Keywords:** misclassification, offset, binary model, risk difference, relative risk, odds ratio

## Abstract

**Highlights:**

**Public health relevance—How does this work relate to a public health issue?**

- Use of unbiased gold-standard measures is limited in public health or epidemiologic research.
- Surrogates or proxy measures are more often used despite their vulnerability to measurement or misclassification errors.

**Public health significance—Why is this work of significance to public health?**

- Misclassification errors induce biases in research results.
- Development of methods for corrections of biases pertinent to given circumstances is thus strived for.

**Public health implications—What are the key implications or messages for practitioners, policy makers and/or researchers in public health?**

- Biased research results may misdirect public health policies.
- Careful assessment of the extent of misclassification errors is critical for unbiased results.

**Abstract:**

In randomized clinical trials and observational studies alike, it is difficult and challenging to collect gold-standard outcome measures for all participants. Although it would be ideal to use gold-standard measures, the costs and logistics of collecting them are often prohibitive. Therefore, surrogate or proxy measures or screening survey instruments are more often used to mitigate such difficulties, yet at the expense of misclassification errors and consequent biased statistical inferences. In this paper, when misclassification errors of proxy measures in comparison to a gold-standard measure are available through external or internal validation samples, we determined appropriate offset values in generalized binary regression models as a function of the proxy measure to eliminate biases of estimated effects in terms of risk difference, relative risk, and odds ratio that are incurred due to misclassification errors. Simulation studies were conducted to empirically demonstrate and verify the approach using appropriate offset values specific to each binary effect measure for estimating unbiased effects. Both point estimates of all effect measures and standard errors of regression coefficients obtained from the proposed offset-adjusted binary models were shown to be unbiased.

## 1. Introduction

Misclassification errors are inevitable in any research area, especially when gold-standard measures are not available or used [1]. Even if gold-standard measures may not be free of misclassification or misdiagnosis errors, depending on research areas, they are often expensive, time-consuming and logistically challenging to collect to an extent that many studies, large-scale studies in particular, may not be able to afford [2]. Therefore, less expensive, more convenient, and reliable proxy or surrogate measures, screening tools, or self-reported survey instrument responses are often instead used for research investigations.

Examples include the diagnosis of various diseases, such as cancer and major depressive disorder (MDD), to mention only a couple. For instance, a gold-standard diagnosis of prostate cancer can be made by biopsy guided by a transrectal ultrasound or MRI imaging [3,4]. A surrogate diagnosis could be made based on the prostate-specific antigen (PSA) level in the blood [5], which can serve as a screening tool. In addition, there are symptom-based self-administered survey instruments for prostate cancer diagnosis, such as the International Prostate Symptom Score (IPSS) [6]. For the diagnosis of MDD, a gold-standard diagnosis can be made through clinical interviews using the Structured Clinical Interview for DSM (SCID) [7,8]. Instead, the symptom-based Hamilton Depression Rating Scale (HDRS) [9] or Patient Health Questionnare-9 (PHQ-9) scale [10] is more often used. After all, none of these surrogate screening instruments may be free of misclassification errors compared with the gold-standard measures. Therefore, statistical inferences based on proxy or self-reported data will suffer biases in effect estimates of exposures, interventions, or treatments on study outcomes [11,12,13].

Numerous epidemiological and statistical approaches have been developed to adjust for misclassification and measurement errors, thereby mitigating potential biases in inferences, as introduced and compiled in several books [14,15,16,17,18]. This reflects the fact that measurement errors are difficult issues to appropriately handle and affect analysis results in broad research fields, including but not limited to epidemiology, public health, and statistics. We attempted to propose a relatively simple and straightforward method to adjust misclassification errors through building appropriate offset values in binary models to estimate unbiased effect sizes in terms of risk difference, relative risk, and odds ratio. The offset values specific to each effect measure will be determined based on the probabilities of misclassification errors of proxy measures compared to a gold-standard measure, which is often available through external or internal validation samples. We believe that the proposed approach to correcting biases through offset adjustment is novel and has not been previously studied. We presented a hypothetical randomized study example below, in which two groups were compared using several different measures of outcomes, and simulation studies were conducted to empirically verify the proposed approach in terms of unbiased point estimates and standard errors.

## 2. Materials and Methods

### 2.1. Setting and Notations

Suppose a randomized clinical trial is designed to compare adequate adherence to a medication for treating a disease between two study arms. The most rigorous and valid way to accurately measure and ascertain adherence would be to directly observe on a daily basis a participant’s ingestion of the medication in the presence of a healthcare professional treating the participant, which is called directly observed therapy (DOT). The daily DOT adherence measure could be considered a gold-standard measure. However, it would be extremely difficult and costly in many circumstances for both the patients and the health care professionals to ingest and observe, respectively, at the same time in a clinic or another appointed place on a daily basis.

To mitigate that kind of logistical burden and difficulties, the study considered dispensing medications in electronic devices that can capture and timestamp the retrieval of medications. For instance, an electronic blister pack containing one or two weeks worth of direct-acting antiviral (DAA) medications for treating hepatitis C virus (HCV) in seven or fourteen blisters can record the day and time when a blister containing a medication was opened up for retrieval of the medication inside [19]. Such blister packs have been used in several studies, including but not limited to SIMPLIFY [20], D3FEAT [21], PREVAIL [22], and HERO study [23]. Although this is also a rigorous way of measuring daily adherence and can serve as a close proxy measure of the DOT gold standard, the electronic blister packs are not able to record or validate whether the participant indeed ingested the retrieved medications. In addition, self-reported adherence was also collected to estimate the extent of validation of self-reported data, which would be the most convenient and least costly way of measuring adherence in this kind of study and beyond.

Suppose that the target binary outcome, i.e., adequate adherence, is declared if more than 75% days during the medication period are adherent to the medication regimen. Let us denote (1) the “true” adequate adherence by Y* when declared based on the directly observed ingestion of medications; (2) a proxy adequate adherence of Y* by Y when declared based on the electronic blister pack records; (3) a convenient proxy adequate adherence of Y* by Z when declared based on the self-reported adherence. An indicator variable representing the two arms is denoted by X.

### 2.2. Assumptions

All measures, Y*, Y and Z, in addition to the random assignment indicator X, are assumed to be scored 0 or 1 on a binary scale. We assume that (A1) P(Z|X) is hypothesized and therefore known; (A2) the study measured both Y and Z and therefore P(Y|Z) is known; (A3) P(Y*|Z) is known from external validation studies or from an internal small validation sample; (A4) conditional probabilities of Y given Z and Y* given Y are both independent of X, i.e., Y|Z⊥X and Y*|Y⊥X by virtue of random assignments of X; that is, P(Y|Z, X) = P(Y|Z) and P(Y*|Y, X) = P(Y*|Y), which is referred to as non-differential misclassification (with respect to X) [24].

Based on these assumptions, derivations of P(Y*|X) and P(Y|X) are possible as follows. For instance, P(Y|X) = P(Y|Z = 0, X)P(Z = 0|X) + P(Y|Z = 1, X)P(Z = 1|X) = P(Y|Z = 0)P(Z = 0|X) + P(Y|Z = 1)P(Z = 1|X) owing to the law of total probability for the first equation and to assumption A4 for the second. Once P(Y|X) is derived this way, P(Y*|X) can similarly be derived based on assumptions A3 and A4.

### 2.3. Effect Measures

The goal of the statistical inference is to build a statistical model concerning P(Y*|X) and thereby estimate the following “true” binary outcome effect measures:

Risk difference (RD) = P(Y* = 1|X = 1) − P(Y* = 1|X = 0);

Risk ratio (RR) = P(Y* = 1|X = 1)/P(Y* = 1|X = 0);

and

Odds ratio (OR) = P(Y* = 1|X = 1)P(Y* = 1|X = 0)/P(Y* = 0|X = 1)/P(Y* = 0|X = 0).

### 2.4. Statistical Models

A generalized linear model for the true binary outcome Y* can be expressed in terms of X as follows:

g(P(Y* = 1|X)) = β_0.g_ + β_1.g_X, (1)

where g(·) is called a link function. Specifically, g(·) = I(·) = ·, called the identity link function, for estimating RD = β_1.g_ = β_1.RD_, g(·) = log(·), called log-link function, for estimating RR = exp(β_1.g_) = exp(β_1.RR_), and g(·) = logit(·) = log(·/(1 − ·)), i.e., log-odds and called logit-link function, for estimating OR = exp(β_1.g_) = exp(β_1.OR_).

Given that Y* is not available for all participants in reality, we attempt to build a generalized linear model for P(Y|X) that will be equivalent to model (1) by constructing appropriate offset values specific to each effect measure above as a function of Z (i.e., free of both Y* and X). Specifically, we attempted to estimate β_1.g_ in model (1) in particular by estimating ξ_1.g_ in the following model for Y with offset_g_(Z) as a function of Z and a link function g(·),

g(P(Y = 1|X, Z)) = ξ_0.g_ + ξ_1.g_X + offset_g_(Z) (2)

where

offset_g_(Z) = ζ_0.g_(1 − Z) + ζ_1.g_Z. (3)

We will compare the performances of this model with model (1) to assess bias of ξ_1.g_ or exp(ξ_1.g_) in comparison with β_1.g_ or exp(β_1.g_), in terms of RD, RR and OR when offset_g_(Z) is appropriately determined.

Model (1) will also be compared with the following models as well to assess bias of effect measures due to misclassification when Y or Z is regressed on X without any adjustment using offset values.

g(P(Z = 1|X)) = φ_0.g_ + φ_1.g_X, (4)

and

g(P(Y = 1|X)) = η_0.g_ + η_1.g_X. (5)

Similar to model (X), when g(·) = I(·), φ_1.g_ = φ_1.RD_ and η_1.g_ = η_1.RD_ for estimating RD; when g(·) = log(·), exp(φ_1.g_) = exp(φ_1.RR_) and exp(η_1.g_) = exp(η_1.RR_) for estimating RR; and when g(·) = logit(·), exp(φ_1.g_) = exp(φ_1.OR_) and exp(η_1.g_) = exp(η_1.OR_) for estimating OR. In the following, let us denote π_0_* = P(Y* = 1|Z = 0), π_1_* = P(Y* = 1|Z = 1), π_0_ = P(Y = 1|Z = 0) and π_1_ = P(Y = 1|Z = 1).

### 2.5. Derivations of Offset Values

#### 2.5.1. Offset for RD Model

For estimating the “true” RD = β_1.RD_, when g(·) = I(·), the offset(Z) can be determined by taking the following steps. First, we consider the following identity link model:

I(P(Y* = 1|Z)) = P(Y* = 1|Z) = δ_0.RD_ + δ_1.RD_Z.

Second, we consider identity link-specific correction factors, ζ_0.RD_ and ζ_1.RD_, depending on Z, so that P(Y* = 1|Z = 0) + ζ_0.RD_ = δ_0.RD_ + ζ_0.RD_ = P(Y = 1|Z = 0) and P(Y* = 1|Z = 1) + ζ_1.RD_ = δ_0.RD_ + δ_1.RD_ + ζ_1.RD_ = P(Y = 1|Z = 1). It follows that

ζ_0.RD_ = π_0_ − π_0_*

and

ζ_1.RD_ = π_1_ − π_1_*.

Therefore, if we determine the identity link-specific offset as

offset_RD_(Z) = ζ_0.RD_(1 − Z) + ζ_1.RD_Z (6)

in the following model

I(P(Y = 1|Z)) = γ_0.RD_ + γ_1.RD_Z + ζ_0.RD_ (1 − Z) + ζ_1.RD_Z,

then we will have γ_0.RD_ = δ_0.RD_ and γ_1.RD_ = δ_1.RD_. Therefore, if we fit the following model,

I(P(Y = 1|X, Z)) = ξ_0.RD_ + ξ_1.RD_X + ζ_0.RD_ (1 − Z) + ζ_1.RD_Z,

then we will have ξ_0.RD_ = β_1.RD_ and ξ_1.RD_ = β_1.RD_.

#### 2.5.2. Offset for RR Model

To estimate the “true” RR = exp(β_1.RR_), when g(·) = log(·), first, we consider the following model:

log(P(Y* = 1|Z)) = δ_0.RR_ + δ_1.RR_Z.

Under this log-link model, we consider log-link-specific correction factors ζ_0.RR_ and ζ_1.RR_, depending on Z so that log(P(Y* = 1|Z = 0)) + ζ_0.RR_ = δ_0.RR_ + ζ_0.RR_ = log(P(Y = 1|Z = 0)) and log(P(Y* = 1|Z = 1)) + ζ_1.RR_ = δ_0.RR_ + δ_1.RR_ + ζ_1.RR_ = log(P(Y = 1|Z = 1)). It follows that

ζ_0.RR_ = log(π_0_/π_0_*)

and

ζ_1.RR_ = log(π_1_/π_1_*).

Therefore, if we determine the log-link specific offset as

offset_RR_(Z) = ζ_0.RR_(1 − Z) + ζ_1.RR_Z (7)

in the following model

log(P(Y = 1|Z)) = γ_0.RR_ + γ_1.RR_Z + ζ_0.RR_ (1 − Z) + ζ_1.RR_Z,

then we will have γ_0.RR_ = δ_0.RR_ and γ_1.RR_ = δ_1.RR_. Therefore, if we fit the following model,

log(P(Y = 1|X, Z)) = ξ_0.RR_ + ξ_1.RR_X + ζ_0.RR_ (1 − Z) + ζ_1.RR_Z,

then we will have ξ_0.RR_ = β_1.RR_ and ξ_1.RR_ = β_1.RR_.

#### 2.5.3. Offset for OR Model

To estimate the “true” OR = exp(β_1.OR_), when g(·) = logit(·), we first consider the following logit-link model

logit(P(Y* = 1|Z)) = δ_0.OR_ + δ_1.OR_Z.

Under this model, we consider logit-link specific correction factors ζ_0.OR_ and ζ_1.OR_, depending on Z, so that logit(P(Y* = 1|Z = 0)) + ζ_0.OR_ = δ_0.OR_ + ζ_0.OR_ = logit(P(Y = 1|Z = 0)) and logit(P(Y* = 1|Z = 1)) + ζ_1.OR_ = δ_0.OR_ + δ_1.OR_ + ζ_1.OR_ = logit(P(Y = 1|Z = 1)). It follows that

ζ_0.OR_ = log(π_0_(1 − π_0_*)/π_0_*/(1 − π_0_))

and

ζ_1.OR_ = log(π_1_(1 − π_1_*)/π_1_*/(1 − π_1_)).

Therefore, if we determine the log-link specific offset as

offset_OR_(Z) = ζ_0.OR_(1 − Z) + ζ_1.OR_Z (8)

in the following model

logit(P(Y = 1|Z)) = γ_0.OR_ + γ_1.OR_Z + ζ_0.OR_ (1 − Z) + ζ_1.OR_Z,

then we will have γ_0.OR_ = δ_0.OR_ and γ_1.OR_ = δ_1.OR_. Therefore, if we fit the following model,

logit(P(Y = 1|X, Z)) = ξ_0.OR_ + ξ_1.OR_X + ζ_0.OR_ (1 − Z) + ζ_1.OR_Z,

then we will have ξ_0.OR_ = β_1.OR_ and ξ_1.OR_ = β_1.OR_. All of these models, regression equations, coefficients, offset functions, and notations are summarized in Table 1 below.

### 2.6. Simulation Studies

We consider 8 scenarios for simulation evaluations of biases of model (2), in particular, in comparison with model (1), with prespecified probability presented in Table 2, which also includes derived probabilities and true effect measures thereof, following the steps described in the Section 2.2 above. For each scenario, we ran 1000 (*i* = 1 to 1000) simulations with N = 500 (*j* = 1 to 500) samples for each simulation. For the *i*-simulation for any scenario, we randomly generated variables in the following sequence: (1) X*_ij_* for the *j*-th participant with a 1:1 or 2:1 ratio between X = 1 and X = 0; (2) Z*_ij_* based on X*_ij_* and the prespecified P(Z|X); (3) Y*_ij_* based on Z*_ij_* and the prespecified P(Y|Z); and (4) Y*_ij_ based on Y*_ij_* and the prespecified P(Y*|Y). With observed data simulated in this way, we estimated observed probabilities of π_0i_*, π_1i_*, π_0*i*_ and π_1*i*_ for the *i*-th simulation based on estimates from N = 500 observations and then determined offset_g_(Z)*_i_* = ζ_0.g*i*_(1 − Z) + ζ_1.g*i*_Z, following Equations (6) for RD, (7) for RR and (8) for OR. We note that in practice, both π_0i_* and π_1i_* would be estimated based on a smaller sample size than N = 500 for internal validation, but here we used all observations to have more accurate estimates for verification purposes. Finally, we fitted model (2) with the observed offsets for the *i*-th simulation. Let us denote estimated effect measures from the *i*-th simulated data as follows: *f*(ξ_1.RD*i*_) = ξ_1.RD*i*_ for RD*_i_*, *f*(ξ_1.RR*i*_) = exp(ξ_1.RR*i*_) for RR*_i_* and *f*(ξ_1.OR*i*_) = exp(ξ_1.OR*i*_) for OR*_i_*. In addition, we also fitted the “true” model (1), and we denote the estimated standard errors obtained from models (1) and (2) by SE(β_1.g*i*_) and SE(ξ_1.g*i*_), respectively, for the *i*-th simulated data set. SAS v9.4 (SAS Inc. Cary, NC, USA) simulation code with proc genmod is provided in the Supplementary File.

### 2.7. Expected and Empirical Bias and %Bias

The *expected* bias of the effect measures estimated based on models (4) and (5) was calculated in comparison to the “true” effect measures in model (1) that are presented in Table 2. For instance, biases were calculated as bias(*f*(φ_1.g_)) = *f*(φ_1.g_) − *f*(β_1.g_) and bias(*f*(η_1.g_)) = *f*(η_1.g_) − *f*(β_1.g_), where *f*(·) = I(·) for RR and *f*(·) = exp(·) for both RR and OR. Subsequently, their corresponding %biases were calculated as %bias(*f*(φ_1.g_)) = bias(*f*(φ_1.g_))/*f*(β_1.g_) × 100%, and %bias(*f*(η_1.g_)) = bias(*f*(η_1.g_))/*f*(β_1.g_) × 100%.

As for the simulation-based *empirical* biases of effect measures of model (2), we first obtained an average of 1000 estimated *f*(ξ_1.g*i*_), say Avg(*f*(ξ_1.g*i*_)), and then bias(*f*(ξ_1.g*i*_)) was calculated as bias(*f*(ξ_1.g_)) = Avg(*f*(ξ_1.g*i*_)) − *f*(β_1.g_) and %bias(*f*(ξ_1.g_)) as %bias(*f*(ξ_1.g_)) = bias(*f*(ξ_1.g_))/*f*(β_1.g_) × 100%. We also compared the *empirical* standard errors (SEs) of the regression coefficients for models (1) and (2), since the SEs were not theoretically derived in closed forms, and thus we estimated them from the simulated data. The biases of SE(ξ_1.g_) in comparison to SE(β_1.g_) were defined as bias(SE(ξ_1.g_)) = Avg(SE(ξ_1.g*i*_)) − Avg(SE(β_1.g*i*_)) and %bias as bias(SE(ξ_1.g_)) = bias(SE(ξ_1.g*i*_))/Avg(SE(β_1.g*i*_)) × 100%.

## 3. Results

### 3.1. Expected Bias of Models Without Offset Adjustment

Table 3 presents the extents of the biases of effects expected from models fitting Z (4) or Y (5) with X alone in comparison to the model fitting Y* with X, the “true” model (1). The effect measures—RD, RR, and OR—were computed based on the prespecified probability, P(Z = 1|X), and derived probabilities. P(Y = 1|X) and P(Y* = 1|X), as presented in Table 2. Across all eight scenarios, compared to the true effect measures in Table 1, %biases of expected effect measures from models fitting Z with X were much greater than those fitting Y with X. Regardless of models and scenarios, however, the sizes of %bias were substantially large, and the %bias was greatest for RD followed by OR and RR, which had the smallest %bias. Specifically, for model (4), the range of %bias RD was 108.3–316.7%, that of RR was 32.7–52.0% and that of OR was 53.1–162.2%. For model (5), the range of %bias RD was 25.0–66.7%, that of RR was 5.0–9.5% and that of OR was 10.2–21.5%. It follows that all effect measures calculated based on models (4) and (5) markedly overestimated the true effect measures calculated based on model (1).

### 3.2. Empirical Bias of Estimated Effect Measures from Models with Offset Adjustment Assessed by Simulations

Table 4 presents simulation-based empirical %biases of effect measures estimated based on model (2) with offset adjustment (3). Across all scenarios, the empirically estimated RD = ξ_1.RD_ tended to overestimate the true RD = β_1.RD_, with %bias ranging from 5.5% to 13.6%. However, the sizes are much smaller by far than the %biases of RDs based on models (4) and (5) presented above in Table 3. In contrast, the %biases of both the empirically estimated RR = exp(ξ_1.RR_) and OR = exp(ξ_1.OR_) are negligible without clear direction of over- or underestimation of the true effect measures of RR = exp(β_1.RR_) and OR = exp(β_1.OR_) obtained from model (1), respectively, ranging from −4.0% to 3.8% for the former and −2.2% to 0.9% for the latter. These %biases are again much smaller compared to the %biases of RRs and ORs based on models (4) and (5), presented above in Table 3.

### 3.3. Empirical Bias of Standard Errors Estimated from Model with Offset (2) in Comparison to Those from the True Model (1)

Table 5 presents simulation-based empirical %biases of SE(ξ_1.g_) of regression coefficients of model (2) with offset adjustment (3). Across all scenarios, the empirically estimated SE(ξ_1.RD_) and SE(ξ_1.OR_) tended to overestimate, albeit negligibly, the SE(β_1.RD_) and SE(β_1.OR_) of the true model (1). However, the direction of the bias(SE(ξ_1.RR_)) depended on the scenario, but the extent of the bias was similarly almost ignorable.

## 4. Discussion

We demonstrated that appropriate determinations of offsets reduced or eliminated biases of binary effect measures—RD, RR, and OR—that incur when proxy or surrogate measures with misclassification errors are modeled and fitted without adjustment of offsets. Although the %bias of RD was not completely eliminated, its absolute bias was <0.018 across all scenarios. On the other hand, %biases of RR and OR were very close to zero, yielding unbiased estimates. The estimated standard errors are also shown to be little biased, regardless of the effect measure. Collectively, inferences about or tests of the significance of any effect measures based on the estimates and standard errors obtained from model (2) would be valid with little bias, regardless of allocation ratios.

Although model (2) was simple, many simulations failed to fit with a log link for RR estimation. Nonetheless, unbiased estimates were yielded based on averages of fitted simulations, as shown in Table 4. The number of failed fits depends on the simulation scenarios (ranging from 5% to 81%), but the underlying reasons are unknown. However, there was no failed fit for estimation of RD despite non-trivial empirical %biases, but their absolute biases appear negligible. No failed fit was observed for the estimation of OR with a logit link, and model (2) yielded unbiased estimates. Therefore, model (2) with the canonical logit-link function would result in the most reliable unbiased estimates of OR in view of the simulation results.

Our approach, while addressing a specific situation with both binary outcomes and exposures, is distinctive from existing approaches such as regression calibration [25], the Berkson error model [26,27], and diverse Bayesian approaches [16]. While these methods may be applicable to broader situations, including studies with continuous multiple covariates, their measurement error assumptions often assume a distributional form for the exposure variable X, whereas our approach addresses misclassification of binary outcome measures. Regardless, to the best of our knowledge, no existing methods have used offset approaches.

It would be more practically useful to determine appropriate offsets when only Z or Y (but not both) is available in studies, and when internal or external validation of either P(Y*|Z) or P(Y*|Y) is available. However, in either case, it is not possible to determine offsets similarly to model (2) and offset values (3). It is fundamentally because an offset must be defined as a function X, which requires “direct” knowledge of P(Y*|X) at every individual level. However, if this is the case, model (1) can be fitted without any adjustment with the offset to estimate “true” binary effect measures.

Although the non-differential misclassification assumption (A4) in particular would be acceptable for randomized studies, it might be relatively strong and unacceptable for observational studies in general unless an instrumental variable mimicking randomization is available, which could be achieved through a Mendelian randomization approach [28] if genetic information is available. In practice, therefore, careful assessments of the validity of assumption (A4) should be carried out to determine and apply the proposed processes for determining offset values to reduce biases. Nonetheless, assumption (4) enables investigators at a study design stage to assess the extent of potential biases based on derived probabilities as presented in Table 1 and Table 2 with hypothesized probabilities of misclassification errors, P(Y*|Y) and P(Y|Z). In addition, the availability of unbiased estimates of both π_0_* and π_1_* would be crucial for obtaining unbiased estimates of effect measures by including offsets in a generalized linear model. However, if the validation sample size is relatively small, the estimates of π_0_* and π_1_* would have wider confidence intervals even if their point estimates are unbiased. In this case, it would be worth conducting sensitivity analyses with varying estimates of π_0_* and π_1_* within the confidence interval. As long as the non-differential assumption holds, the offset-adjusted models will yield unbiased estimates of the effects and standard errors of the regression coefficients corresponding to each of the varying estimates.

There are several potential extensions that deserve further study. First, when the non-differential misclassification assumption (A4) is violated, its impact on estimates from the offset-adjusted model (2) can be examined. If the impact is sizable, depending on the extent of the violation or deviation, it would be worth examining whether further adjusted offset functions specific to the combination of at least X and Z would yield unbiased estimates. In addition, estimates of the conditional probabilities, such as P(Y*|X, Z), would be required, perhaps again based on external or internal validation samples. Second, it will be helpful to extend the approaches to determining offset values as a future study when covariates (let us say W collectively) should be included in model (1) and (2) adjustments. For instance, it should be examined what kind of assumptions concerning relationships of W with other measures should be made, whether offsets should be a function of W as well, and lastly, whether it would ever be possible to determine appropriate offsets in the presence of W in a model. Third, extension of the proposed approach to studies that compare more than two groups would also represent an interesting future study and so would be the extension of the proposed approach to ordinal or nominal outcomes with >2 categories. Fourth, it is unknown in this study why RD shows a larger % bias. Therefore, large- or finite-sample theoretical derivations would be needed to derive asymptotic or finite-sample properties of the RD (and RR and OR as well) estimate under the offset-adjusted models to examine this issue. Lastly, applying the proposed methods to real data analysis would be more illustrative.

## 5. Conclusions

In conclusion, biases of binary effect measures can be substantial when misclassification errors are unavoidable. However, the biases could be reduced or eliminated by including appropriate offset values in generalized binary regression models. The proposed approach also yields unbiased standard errors of regression coefficients and has potential applicability across diverse areas of the health sciences and beyond, helping mitigate concerns about inferential biases and the significance testing of estimated effects of interventions, treatments, or exposures due to misclassification errors in binary outcomes.

## Figures and Tables

**Table 1:** Summary of models, regression equations, regression coefficients, offset functions, and notations.

Model for “true” P(Y*|X): g(P(Y* = 1|X)) = β_0.g_ + β_1.g_X Equation (1)			Model for Misclassified P(Z|X): g(P(Z = 1|X)) = φ_0.g_ + φ_1.g_X, Equation (4)			Model for Misclassified P(Y|X): g(P(Y = 1|X)) = η_0.g_ + η_1.g_X, Equation (5)	
**Regression** **Equation**	**Effect** **Estimate**		**Regression** **Equation**		**Effect** **Estimate**	**Regression** **Equation**	**Effect** **Estimate**
I(P(Y* = 1|X)) = β_0.RD_ + β_1.RD_X	RD = β_1.RD_		I(P(Z = 1|X)) = φ_0.RD_ + φ_1.RD_X		RD = φ_1.RD_	I(P(Y* = 1|X)) = η_0.RD_ + η_1.RD_X	RD = η_1.RD_
log(P(Y* = 1|X)) = β_0.RR_ + β_1.RR_X	RR = exp(β_1.RR_)		log(P(Y* = 1|X)) = φ_0.RR_ + φ_1.RR_X		RR = exp(φ_1.RR_)	log(P(Y* = 1|X)) = η_0.RR_ + η_1.RR_X	RR = exp(η_1.RR_)
logit(P(Y* = 1|X)) = β_0.OR_ + β_1.OR_X	OR = exp(β_1.OR_)		logit(P(Y* = 1|X)) = φ_0.OR_ + φ_1.OR_X		OR = xp(φ_1.OR_)	logit(P(Y* = 1|X)) = η_0.OR_ + η_1.OR_X	OR = exp(η_1.OR_)
Offset-Adjusted Model: g(P(Y = 1|X, Z)) = ξ_0.g_ + ξ_1.g_X + Offset_g_(Z), Equations (2) and (3)							
**Regression Equation**		**Effect** **Estimate**		**Offset_g_(Z)**		**Offset_g_(Z) Coefficients**	
I(P(Y = 1|X, Z)) = ξ_0.RD_ + ξ_1.RD_X + offset_RD_(Z)		RD = ξ_1.RD_		offset_RD_(Z) = ζ_0.RD_(1 − Z) + ζ_1.RD_Z, Equation (6)		ζ_0.RD_ = π_0_ − π_0_ *, ζ_1.RD_ = π_1_ − π_1_*	
log(P(Y = 1|X, Z)) = ξ_0.RR_ + ξ_1.RR_X + offset_RR_(Z)		RR = exp(ξ_1.RR_)		offset_RR_(Z) = ζ_0.RR_(1 − Z) + ζ_1.RR_Z, Equation (7)		ζ_0.RR_ = log(π_0_/π_0_*), ζ_1.RR_ = log(π_1_/π_1_*)	
logit(P(Y = 1|X, Z)) = ξ_0.OR_ + ξ_1.OR_X + offset_OR_(Z)		OR = exp(ξ_1.OR_)		offset_OR_(Z) = ζ_0.OR_(1 − Z) + ζ_1.OR_Z, Equation (8)		ζ_0.OR_ = log(π_0_(1 − π_0_*)/π_0_*/(1 − π_0_)), ζ_1.OR_ = log(π_1_(1 − π_1_*)/π_1_*/(1 − π_1_))	

Note: π_0_* = P(Y* = 1|Z = 0), π_1_* = P(Y* = 1|Z = 1), π_0_ = P(Y = 1|Z = 0) and π_1_ = P(Y = 1|Z = 1).

**Table 2:** Prespecified simulation input probabilities and derived probabilities and true effect measure from thereof with both 1:1 and 2:1 random allocation between X = 0 and = 1, i.e., P(X = 1) = P(X = 0) = ½ for the former and P(X = 1) = 2/3 and P(X = 0) = 1/3.

	Prespecified Simulation Input Probabilities						Derived Probabilities				True Effects		
Scenario	P(Z = 1|X)		P(Y = 1|Z)		P(Y* = 1|Y)		P(Y = 1|X)		P(Y* = 1|X)		RD	RR	OR
	X = 0	X = 1	Z = 0	Z = 1	Y = 0	Y = 1	X = 0	X = 1	X = 0	X = 1	β_1.RD_	exp(β_1.RR_)	exp(β_1.OR_)
1	0.4	0.7	0.2	0.8	0.1	0.9	0.44	0.62	0.452	0.596	0.144	1.319	1.789
2	0.4	0.7	0.3	0.7	0.2	0.8	0.46	0.58	0.476	0.548	0.072	1.151	1.335
3	0.4	0.6	0.2	0.8	0.1	0.9	0.44	0.56	0.452	0.548	0.096	1.212	1.470
4	0.4	0.6	0.3	0.7	0.2	0.8	0.46	0.54	0.476	0.524	0.048	1.101	1.212
5	0.3	0.6	0.2	0.8	0.1	0.9	0.38	0.56	0.404	0.548	0.144	1.356	1.789
6	0.3	0.6	0.3	0.7	0.2	0.8	0.42	0.54	0.452	0.524	0.072	1.159	1.335
7	0.3	0.5	0.2	0.8	0.1	0.9	0.38	0.50	0.404	0.500	0.096	1.238	1.475
8	0.3	0.5	0.3	0.7	0.2	0.8	0.42	0.50	0.452	0.500	0.048	1.106	1.212

**Table 3:** Expected biases and %biases of estimates obtained based on P(Z = 1|X) and P(Y = 1|X) in comparison with true effects presented in Table 2.

		Effects Based on P(Z = 1|X)			Effects Based on P(Y = 1|X)		
	Model	g(P(Z = 1|X)) = φ_0_ + φ_1_X, Equation (4)			g(P(Y = 1|X)) = η_0_ + η_1_X, Equation (5)		
	Link g(·)	I(·)	log(·)	logit(·)	I(·)	log(·)	logit(·)
Scenario	Effect Measure	RD = φ_1.RD_	RR = exp(φ_1.RR_)	OR = exp(φ_1.OR_)	RD = η_1.RD_	RR = exp(η_1.RR_)	OR = exp(η_1.OR_)
1	Bias	0.156	0.431	1.711	0.036	0.091	0.288
	%Bias	108.3%	32.7%	95.7%	25.0%	6.9%	16.1%
2	Bias	0.228	0.599	2.165	0.048	0.110	0.286
	%Bias	316.7%	52.0%	162.2%	66.7%	9.5%	21.5%
3	Bias	0.104	0.288	0.780	0.024	0.060	0.150
	%Bias	108.3%	23.7%	53.1%	25.0%	5.0%	10.2%
4	Bias	0.152	0.399	1.038	0.032	0.073	0.166
	%Bias	316.7%	36.3%	85.7%	66.7%	6.6%	13.7%
5	Bias	0.156	0.644	1.711	0.036	0.117	0.288
	%Bias	108.3%	47.4%	95.7%	25.0%	8.6%	16.1%
6	Bias	0.228	0.841	2.165	0.048	0.126	0.286
	%Bias	316.7%	72.5%	162.2%	66.7%	10.9%	21.5%
7	Bias	0.104	0.429	0.858	0.024	0.078	0.156
	%Bias	108.3%	34.7%	58.2%	25.0%	6.3%	10.6%
8	Bias	0.152	0.560	1.121	0.032	0.084	0.169
	%Bias	316.7%	50.7%	92.5%	66.7%	7.6%	13.9%

**Table 4:** Estimated effects based on an offset-adjusted model g(P(Y = 1|X, Z)) = ξ_0_ + ξ_1_X + offset(Z), Equations (2) and (3), and its biases and %biases empirically assessed in comparison with true effects based on sample size of N = 500 and 1000 simulations for each scenario.

Model	g(P(Y = 1|X, Z)) = ξ_0_ + ξ_1_X + Offset(Z), Equations (2) and (3)									
Link	g(·) = I(·)			g(·) = log(·)				g(·) = logit(·)		
Effect	RD = ξ_1.RD_			RR = exp(ξ_1.RR_)				OR = exp(ξ_1.OR_)		
Scenario	Estimate	Bias	%Bias	Estimate	Bias	%Bias	%Failure *	Estimate	Bias	%Bias
**1:1 Balanced Allocation Between X = 1 and X = 0**										
1	0.162	0.018	12.4%	1.266	−0.053	−4.0%	5%	1.755	−0.034	−1.9%
2	0.077	0.005	7.3%	1.151	0.000	0.0%	55%	1.347	0.012	0.9%
3	0.109	0.013	13.6%	1.195	−0.018	−1.5%	4%	1.456	−0.013	−0.9%
4	0.050	0.002	4.5%	1.107	0.007	0.6%	54%	1.214	0.002	0.2%
5	0.160	0.016	11.4%	1.358	0.001	0.1%	25%	1.750	−0.039	−2.2%
6	0.076	0.004	5.5%	1.203	0.044	3.8%	80%	1.337	0.003	0.2%
7	0.109	0.013	13.2%	1.266	0.028	2.3%	26%	1.474	−0.001	−0.1%
8	0.053	0.005	10.6%	1.144	0.038	3.5%	81%	1.232	0.020	1.7%
**2:1 Unbalanced Allocation Between X = 1 and X = 0**										
1	0.158	0.014	9.4%	1.265	−0.054	−4.1%	7%	1.732	−0.056	−3.1%
2	0.078	0.006	8.5%	1.160	0.008	0.7%	53%	1.352	0.017	1.3%
3	0.108	0.012	13.0%	1.199	−0.013	−1.1%	8%	1.457	−0.013	−0.9%
4	0.052	0.004	8.0%	1.114	0.013	1.2%	54%	1.226	0.014	1.1%
5	0.162	0.018	12.3%	1.373	0.017	1.3%	28%	1.764	−0.025	−1.4%
6	0.075	0.003	4.7%	1.200	0.041	3.5%	79%	1.338	0.003	0.2%
7	0.108	0.012	13.0%	1.268	0.031	2.5%	26%	1.474	−0.001	−0.1%
8	0.052	0.004	8.2%	1.158	0.052	4.7%	79%	1.232	0.019	1.6%

* %Failure: Proportion of failed fitting out N = 1000 simulations.

**Table 5:** Empirical %bias of SE(ξ_1.g_) in comparison to SE(β_1.g_) based on sample size of N = 500 and 1000 simulations for each scenario.

Model	g(P(Y* = 1|X)) = β_0.g_ + β_1.g_X, Equation (1) vs. g(P(Y = 1|X, Z)) = ξ_0_ + ξ_1_X + offset(Z), Equation (2)								
Link	g(·) = I(·) for RD			g(·) = log(·) for RR			g(·) = logit(·) for OR		
Scenario	SE(β_1.RD_)	SE(ξ_1.RD_)	%Bias	SE(β_1.RR_)	SE(ξ_1.RR_)	%Bias	SE(β_1.OR_)	SE(ξ_1.OR_)	%Bias
**1:1 Balanced Allocation Between X = 1 and X = 0**									
1	0.044	0.046	4.5%	0.087	0.084	−3.4%	0.182	0.185	2.2%
2	0.045	0.045	2.2%	0.088	0.085	−3.4%	0.180	0.184	2.2%
3	0.044	0.047	4.5%	0.091	0.088	−2.2%	0.180	0.183	1.7%
4	0.045	0.046	2.2%	0.090	0.088	−2.2%	0.180	0.183	1.7%
5	0.044	0.046	4.5%	0.097	0.097	0.0%	0.182	0.185	2.2%
6	0.045	0.045	2.2%	0.093	0.093	0.0%	0.180	0.184	2.2%
7	0.044	0.046	4.5%	0.100	0.101	1.0%	0.181	0.185	2.2%
8	0.045	0.045	2.2%	0.095	0.096	1.1%	0.180	0.184	2.2%
**2:1 Unbalanced Allocation Between X = 1 and X = 0**									
1	0.047	0.049	4.3%	0.097	0.095	−2.1%	0.192	0.196	2.1%
2	0.047	0.048	2.1%	0.097	0.094	−3.1%	0.191	0.195	2.1%
3	0.047	0.050	4.3%	0.100	0.098	−2.0%	0.191	0.195	1.6%
4	0.047	0.048	2.1%	0.098	0.096	−2.0%	0.191	0.194	2.1%
5	0.047	0.048	4.3%	0.108	0.109	0.9%	0.193	0.197	2.1%
6	0.047	0.048	2.1%	0.102	0.102	0.0%	0.191	0.195	2.1%
7	0.047	0.049	4.3%	0.110	0.112	1.8%	0.193	0.197	2.1%
8	0.047	0.048	2.1%	0.103	0.104	1.0%	0.191	0.195	2.1%

## Data Availability

All data generated or analyzed during this study are included in this published article and its Supplementary Information File. Full reusable SAS v9.4 code for running the simulation is provided in the Supplementary Materials section.

## Supplementary Materials

The following supporting information can be downloaded at https://www.mdpi.com/article/10.3390/ijerph23020220/s1, File S1: SAS simulation code.

## References

[1] Pham A., Cummings M., Lindeman C., Drummond N., Williamson T. (2019). Recognizing misclassification bias in research and medical practice. Fam. Pract..

[2] Umemneku Chikere C.M., Wilson K., Graziadio S., Vale L., Allen A.J. (2019). Diagnostic test evaluation methodology: A systematic review of methods employed to evaluate diagnostic tests in the absence of gold standard—An update. PLoS ONE.

[3] Ahdoot M., Wilbur A.R., Reese S.E., Lebastchi A.H., Mehralivand S., Gomella P.T., Bloom J., Gurram S., Siddiqui M., Pinsky P. (2020). MRI-Targeted, Systematic, and Combined Biopsy for Prostate Cancer Diagnosis. N. Engl. J. Med..

[4] Ahmed H.U., Bosaily A.E.S., Brown L.C., Gabe R., Kaplan R., Parmar M.K., Collaco-Moraes Y., Ward K., Hindley R.G., Freeman A. (2017). Diagnostic accuracy of multi-parametric MRI and TRUS biopsy in prostate cancer (PROMIS): A paired validating confirmatory study. Lancet.

[5] Carter H.B. (2018). Prostate-Specific Antigen (PSA) Screening for Prostate Cancer: Revisiting the Evidence. JAMA.

[6] Roehrborn C.G., Kaplan S.A., Jones J.S., Wang J.T., Bavendam T., Guan Z.H. (2009). Tolterodine Extended Release with or Without Tamsulosin in Men with Lower Urinary Tract Symptoms Including Overactive Bladder Symptoms: Effects of Prostate Size. Eur. Urol..

[7] Spitzer R.L., Williams J.B.W., Gibbon M., First M.B. (1992). The Structured Clinical Interview for DSM-III-R (SCID): I: History, Rationale, and Description. Arch. Gen. Psychiatry.

[8] First M.B., Williams J.B.W., Karg R.S., Spitzer R.L. (2015). Structured Clinical Interview for DSM-5 Disorders, Research Version (SCID-5-RV).

[9] Hamilton M. (1960). A rating scale for depression. J. Neurol. Neurosurg. Psychiatry.

[10] Kroenke K., Spitzer R.L., Williams J.B. (2001). The PHQ-9: Validity of a brief depression severity measure. J. Gen. Intern. Med..

[11] Brenner H., Savitz D.A., Gefeller O. (1993). The effects of joint misclassification of exposure and disease on epidemiologic measures of association. J. Clin. Epidemiol..

[12] Rothman K.J., Greenland S., Lash T.L. (2008). Modern Epidemiology.

[13] Höfler M. (2005). The effect of misclassification on the estimation of association: A review. Int. J. Methods Psychiatr. Res..

[14] Yi G.Y. (2017). Statistical Analysis with Measurement Error or Misclassification: Strategy, Method and Application.

[15] Buonaccorsi J.P. (2010). Measurement Error: Models, Methods, and Applications.

[16] Carroll R.J., Ruppert D., Stefanski L.A., Crainiceanu C.M. (2006). Measurement Error in Nonlinear Models: A Modern Perspective.

[17] Fuller W.A. (1987). Measurement Error Models.

[18] Yi G.Y., Delaigle A., Gustafson P. (2021). Handbook of Measurement Error Models.

[19] Adje Y.H., Brooks K.M., Castillo-Mancilla J.R., Wyles D.L., Anderson P.L., Kiser J.J. (2022). The use of technology-based adherence monitoring in the treatment of hepatitis C virus. Ther. Adv. Infect. Dis..

[20] Grebely J., Dalgard O., Conway B., Cunningham E.B., Bruggmann P., Hajarizadeh B., Amin J., Bruneau J., Hellard M., Litwin A.H. (2018). Sofosbuvir and velpatasvir for hepatitis C virus infection in people with recent injection drug use (SIMPLIFY): An open-label, single-arm, phase 4, multicentre trial. Lancet Gastroenterol. Hepatol..

[21] Grebely J., Conway B., Cunningham E.B., Fraser C., Moriggia A., Gane E., Stedman C., Cooper C., Castro E., Schmid P. (2018). Paritaprevir, ritonavir, ombitasvir, and dasabuvir with and without ribavirin in people with HCV genotype 1 and recent injecting drug use or receiving opioid substitution therapy. Int. J. Drug Policy.

[22] Akiyama M.J., Norton B.L., Arnsten J.H., Agyemang L., Heo M., Litwin A.H. (2019). Intensive Models of Hepatitis C Care for People Who Inject Drugs Receiving Opioid Agonist Therapy: A Randomized Controlled Trial. Ann. Intern. Med..

[23] Litwin A.H., Lum P.J., Taylor L.E., Mehta S.H., Tsui J.I., Feinberg J., Kim A.Y., Norton B.L., Heo M., Arnsten J. (2022). Patient-centred models of hepatitis C treatment for people who inject drugs: A multicentre, pragmatic randomised trial. Lancet Gastroenterol. Hepatol..

[24] Jurek A.M., Greenland S., Maldonado G., Church T.R. (2005). Proper interpretation of non-differential misclassification effects: Expectations vs. observations. Int. J. Epidemiol..

[25] Spiegelman D., McDermott A., Rosner B. (1997). Regression calibration method for correcting measurement-error bias in nutritional epidemiology. Am. J. Clin. Nutr..

[26] Berkson J. (1950). Are there two regressions?. J. Am. Stat. Assoc..

[27] Haber G., Sampson J., Graubard B. (2021). Bias due to Berkson error: Issues when using predicted values in place of observed covariates. Biostatistics.

[28] Sanderson E., Glymour M.M., Holmes M.V., Kang H., Morrison J., Munafò M.R., Palmer T., Schooling C.M., Wallace C., Zhao Q. (2022). Mendelian randomization. Nat. Rev. Methods Primers.

